# Anästhesie bei einer thoraxchirurgischen Patientin mit kongenitaler Muskeldystrophie Typ Ullrich

**DOI:** 10.1007/s00101-022-01124-9

**Published:** 2022-05-30

**Authors:** Thomas Hachenberg, Thorsten Walles, Eva Lücke, Thomas Schilling

**Affiliations:** 1 Universitätsklinik für Anästhesiologie und Intensivtherapie, Universitätsklinikum Magdeburg A.ö.R. Otto-von-Guericke-Universität Magdeburg, Leipziger Str. 44, Magdeburg, Deutschland; 2 Universitätsklinik für Herz- und Thoraxchirurgie, Universitätsklinikum Magdeburg A.ö.R. Otto-von-Guericke-Universität Magdeburg, Magdeburg, Deutschland; 3 Universitätsklinik für Pneumologie, Universitätsklinikum Magdeburg A.ö.R. Otto-von-Guericke-Universität Magdeburg, Magdeburg, Deutschland

**Keywords:** Kongenitale Muskeldystrophie Typ Ullrich, Kollagen VI Mangel, Videoassistierte thorakoskopische Chirurgie, Allgemeinanästhesie, Atemwegsmanagement, Ullrich congenital muscular dystrophy, Collagen VI deficiency, Video-assisted thoracoscopic surgery, General anesthesia, Airway management

## Abstract

Die kongenitale Muskeldystrophie Typ Ullrich (UCMD) ist eine seltene Erkrankung. Weltweit wurden bislang 50 Fälle genetisch gesichert. Autosomal-dominante und rezessive Mutationen des *COL6A1*/*COL6A2* im Chromosom 21q22.3 oder des *COL6A3* im Chromosom 2q37.3 führen zu einem Mangel an Kollagen VI. Typische Merkmale der UCMD sind Muskelschwäche von Körperstamm und Extremitäten, Hyperflexibilität der distalen und Kontrakturen der proximalen Gelenke, Rollstuhlpflichtigkeit im Alter von 9 bis 11 Jahren, Versteifung und Skoliose der Wirbelsäule und eine progrediente restriktive Ventilationsstörung. Etwa 50 % der Kinder benötigen im Alter von 11 bis 12 Jahren eine nichtinvasive Ventilation (NIV), wozu auch eine gestörte Funktion des Diaphragmas beiträgt. Es wird über die Narkose bei einer 21-jährigen Patientin mit einer UCMD berichtet, die seit dem 6. Lebensjahr rollstuhlpflichtig war und bei der seit 2018 eine lebenserhaltene NIV erfolgte. Wegen einer subpleuralen Einblutung in den linken Lungenunterlappen nach Entlastung eines Pneumothorax wurde eine videoassistierte thorakoskopische Chirurgie (VATS) vorgenommen. Die spezifischen Anforderungen durch die UCMD, das Atemwegsmanagement für die Einlungenventilation sowie Aspekte zur Auswahl der Anästhetika werden diskutiert. Nach erfolgreicher VATS konnte die Patientin am 7. postoperativen Tag in die Häuslichkeit entlassen werden.

## Abstract (en)

Ullrich congenital muscular dystrophy (UCMD) is a rare disease caused by mutations in the *COL6A1*, *COL6A2* or *COL6A3* genes leading to deficiency of collagen VI in extracellular matrices (ECM). Patients present with generalized muscle weakness, predominantly in the trunk and proximal limbs, hyperlaxity of distal joints, spinal rigidity, scoliosis and various proximal joint contractures, loss of ambulation by 9–11 years of age and progressive respiratory dysfunction. About 50% of the patients require noninvasive ventilation (NIV) by the age of 11–12 years. We report about a female patient (age 21 years) with severe UCMD. After decompression of spontaneous pneumothorax, a major subpleural hematoma of the left lower lobe emerged necessitating video-assisted thoracoscopic surgery (VATS). Anesthesiological aspects, including underlying disease, comorbidities, airway management for one-lung ventilation and choice of anesthetics for patients with muscular dystrophy are discussed. The clinical course during anesthesia, surgery and postoperatively was uneventful and the patient was discharged 7 days after VATS.

## Einleitung

Kongenitale Muskeldystrophien sind seltene Krankheitsbilder und stellen erhebliche Anforderungen an das anästhesiologische Vorgehen [1]. Dies gilt insbesondere für die videoassistierte thorakoskopische Chirurgie (VATS), die ein spezielles Atemwegsmanagement erfordern [2]. Neben der Myopathie müssen die pulmonale und Begleiterkrankungen des Patienten, Dringlichkeit, Art und Umfang des chirurgischen Eingriffs und mögliche Kontraindikationen gegen Anästhetika berücksichtigt werden. Im vorliegenden Fall wird über eine 21-jährige Patientin mit einer Kollagen-VI-assoziierten Muskeldystrophie berichtet, bei der wegen eines ausgedehnten intrapulmonalen Hämatoms eine VATS erfolgte.

## Fallbericht

Bei der Patientin (Alter 21 Jahre) lag eine kongenitale Muskeldystrophie Typ Ullrich (UCMD) vor, die im Krankheitsverlauf zu hochgradiger Tetraparese und axialer Parese, „Rigid-spine“-Phänomen und schweren Kontrakturen der Extremitäten geführt hatte. Wegen der rechtskonkaven Kyphoskoliose war 2013 eine Wirbelsäulenaufrichtung erfolgt; das Fremdmaterial musste infolge einer Infektion 2018 wieder entfernt werden. Bei hochgradiger Dysphagie wurde die Patientin seit 2013 über eine perkutane endoskopische Jejunostomie enteral ernährt, wies jedoch eine ausgeprägte Kachexie (Körpergröße 150 cm, Körpergewicht 28 kg, BMI 12,5 kg/m^2^) auf. Aktuell bot sie eine starke Einschränkung der Mundöffnung (< 3 cm), einen Schiefhals mit verminderter Reklination, eine symptomatische arterielle Verschlusskrankheit und eine neuromuskuläre Dysfunktion des Mastdarms und der Blase. Bei progredienter respiratorischer Insuffizienz erfolgte seit 2014 eine häusliche nichtinvasive Ventilation (NIV) und seit 2018 eine lebenserhaltende NIV (24 h/Tag).

**Figure 1:**
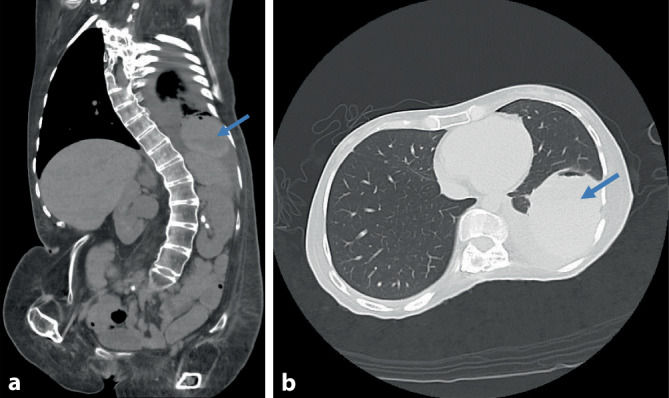

Bei der Patientin waren in der Häuslichkeit akute Schmerzen im linken Thorax mit Ausstrahlung in die Nierenregion eingetreten, weswegen sie vom Rettungsdienst in die zentrale Notaufnahme transportiert wurde. Die Diagnostik ergab einen Pneumothorax links mit maximaler Saumbreite bis zu 1,5 cm bei stabilen Herz-Kreislauf-Verhältnissen und einem kompensierten Gasaustausch unter dauerhafter NIV, sodass zunächst eine konservative Behandlung und Überwachung der Patientin erfolgten [3]. Im weiteren Verlauf vergrößerte sich der Pneumothorax unter der NIV, worauf eine Entlastung mittels Punktion vorgenommen wurde. Darunter entfaltete sich die linke Lunge vollständig, es entwickelten sich jedoch eine ausgedehnte subpleurale Einblutung im Bereich des linken Lungenunterlappens (Abb. 1a,b) und ein Begleiterguss, welcher mehrfach punktiert wurde. Wegen starker Beeinträchtigung der NIV wurden die Indikationen zur VATS und zur Ausräumung des Hämatoms gestellt, der Versuch einer fiberoptischen Wachintubation vor der Narkoseeinleitung musste jedoch wegen schwerer Dyspnoe und kardiopulmonaler Dekompensation der Patientin erfolglos abgebrochen werden. Für den geplanten thoraxchirurgischen Eingriff wurde daher mit ihr eine Allgemeinanästhesie mit der möglichen Notwendigkeit einer Tracheotomie besprochen. Da das Sprechvermögen die einzig verbliebene Möglichkeit der Kommunikation mit den Angehörigen war, sollte eine Tracheotomie nur bei vitaler Indikation vorgenommen werden.

**Figure 2:**
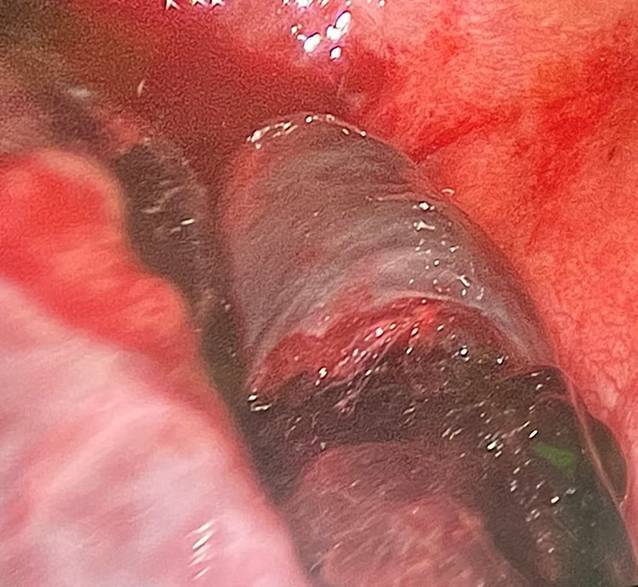

Vor Narkoseeinleitung wurde die NIV durch eine manuelle Maskenbeatmung mit einer FiO_2_ von 1,0 ersetzt, was die Patientin gut tolerierte. Aufgrund der vorbestehenden Muskelerkrankung erfolgte die Narkoseeinleitung mit Propofol (insgesamt 80 mg), Sufentanil (10 µg) und Remifentanil (0,2 µg/kgKG*min). Wegen fehlender Daten zur Wirkung von nichtdepolarisierenden Muskelrelaxanzien bei UCMD und zur weitgehenden Aufrechterhaltung der verbliebenen Muskelkraft wurde auf eine Relaxierung verzichtet. Die Maskenbeatmung gelang komplikationslos, unter tiefer i.v.-Narkose konnte mittels Videolaryngoskopie eine endotracheale Intubation mit einem Einlumentubus (ID 7,0 mm) vorgenommen werden. Die Narkose wurde unter kontrollierter Beatmung als totale intravenöse Anästhesie mit Remifentanil (0,15–0,3 µg/kgKG*min) und Propofol (4 mg/kgKG und h) unter BIS-Monitoring fortgeführt. Nach bronchoskopischer Lagekontrolle des Tubus erfolgte die Anlage eines Bronchusblockers (9F Fuji Uniblocker™, Fuji Systems Co., Ltd., Tokyo, Japan) und Etablierung einer Einlungenventilation (Abb. 2). Intraoperativ bestanden stabile Herz-Kreislauf-Verhältnisse und ein adäquater Gasaustausch ohne Hinweise auf eine maligne Hyperthermie. Die VATS mit Ausräumung des Hämatoms konnte komplikationslos erfolgen; postoperativ wurde die Patientin intubiert und beatmet auf die Intensivstation verlegt, wo sie nach 6 h von der Beatmung entwöhnt und extubiert werden konnte. Die NIV wurde kontinuierlich fortgeführt; es gab keine Hinweise auf eine Verschlechterung der Atemmuskulatur. Zur Schmerztherapie erhielt sie gewichtsadaptiert Piritramid i.v. als Bolus. Der weitere Verlauf war unauffällig; nach Verlegung auf die Normalstation und Entfernung der Thoraxdrainagen wurde die Patientin am 7. postoperativen Tag in die Häuslichkeit entlassen.

## Diskussion

Die UCMD ist die häufigste kongenitale Muskeldystrophie (CMD) nach der merosindefizienten CMD (MDC1A) in Europa, der Fukuyama-CMD in Japan und den α‑Dystroglykanopathien in Australien. Sie wurde 1930 vom deutschen Kinderarzt Otto Ullrich als „kongenitale, atonisch-sklerotische Muskeldystrophie“ und „weiterer Typus der heredodegenerativen Erkrankungen des neuromuskulären Systems“ beschrieben [4, 5]. Die Prävalenz wird mit 1–9/1.000.000 angegeben, laut *Orphanet* wurden weltweit 50 Fälle genetisch gesichert. Autosomal-dominante und rezessive Mutationen des *COL6A1/COL6A2* im Chromosom 21q22.3 oder des *COL6A3* im Chromosom 2q37.3 sind die Ursache der UCMD wie auch der 1976 beschriebenen Bethlem-Myopathie [6, 7]. Das klinische Bild wird wesentlich vom Mangel an Kollagen VI in verschiedenen Geweben beeinflusst, weshalb auch der Terminus Kollagen-VI-assoziierte Myopathie gebräuchlich ist [8]. Kollagen VI ist eine Komponente der extrazellulären Matrix (ECM), wichtig für die strukturelle Verbindung zwischen ECM, Muskeln und Bindegewebe und hat zytoprotektive und -regulierende Eigenschaften [9]. Kollagen VI ist stark vertreten in der ECM von Muskeln, Haut, Sehnen, Knorpel, intervertebralen Bandscheiben, Lungenparenchym, Blutgefäßen und Fettgewebe. Der Mangel führt zu mitochondrialer Dysfunktion, unzureichender Autophagie und gesteigerter Apoptose und daraus resultierenden Funktionsstörungen in besonders betroffenen Strukturen wie Muskeln und Sehnen [10]. Im Gegensatz zu anderen Muskeldystrophien ist bei UCMD die Aktivität der Serumkreatinkinase normal oder nur leicht erhöht. Differenzialdiagnostisch müssen die *RYR1*-assoziierte Myopathie, einschließlich der „central core disease“ und „multiminicore disease“, verschiedene Typen der CMD, die spinale Muskelatrophie und Formen des Ehlers-Danlos- und Marfan-Syndroms abgegrenzt werden. Subtypen der CMD wie die MDC1A, das Walker-Warburg-Syndrom, die Muskel-Auge-Gehirn-Krankheit und die Fukuyama-CMD kommen ebenfalls infrage, wobei bei diesen Krankheitsbildern die mentale Retardierung ein Hauptmerkmal darstellt und die Lebenserwartung deutlich verkürzt ist [8].

Patienten mit UCMD zeigen eine Muskelschwäche von Körperstamm und proximalen Extremitäten, die sich postnatal langsam entwickelt und typischerweise zur Rollstuhlpflichtigkeit im Alter von 10,7 ± 4,8 Jahren führt [7]. Die geistige Entwicklung der Kinder ist normal. Ein charakteristisches Merkmal ist die Hyperflexibilität der distalen Gelenke, im weiteren Verlauf treten häufig eine Versteifung und Skoliose der Wirbelsäule und Kontrakturen der proximalen Gelenke auf. Eine Abnahme der forcierten Vitalkapazität um 6,6 ± 1,9 % pro Jahr wurde bei Kindern im Alter von 6 bis 10 Jahren beschrieben, danach verläuft die Reduktion der Lungenvolumina weniger ausgeprägt [11]. Eine respiratorische Insuffizienz ist postnatal selten, entwickelt sich aber häufig mit zunehmender Krankheitsdauer. Ein frühes Zeichen ist die nächtliche Hypoventilation, sodass die Polysomnographie ein wichtiges diagnostisches Verfahren darstellt. Etwa 50 % der Kinder benötigen bereits im Alter von 11,2 ± 3,6 Jahren eine NIV, wozu neben der restriktiven Ventilationsstörung auch gestörte Funktionen des Diaphragmas und der Atemhilfsmuskulatur beitragen [12]. Nach Initiierung der NIV bleibt die respiratorische Situation häufig über mehrere Jahre stabil. Im vorliegenden Fall war das Krankheitsbild stark ausgeprägt, die Patientin seit dem 6. Lebensjahr rollstuhlpflichtig und wurde seit 2018 lebenserhaltend mit einer NIV behandelt. Die Indikation zur VATS ergab sich durch die zunehmende Verschlechterung der Lungenfunktion infolge der subpleuralen Einblutung, die nicht durch eine Thoraxdrainage entlastet werden konnte.

Aus anästhesiologischer Sicht sind bei Patienten mit UCMD verschiedene Aspekte zu berücksichtigen. Die Skoliose und Kontrakturen der Extremitäten sowie auch der häufig stark eingeschränkte körperliche Zustand erfordern eine sorgfältige Lagerung auf dem OP-Tisch zur Vermeidung von Gesundheitsschäden wie Dekubitalulzera oder Nervenläsionen. Durch die eingeschränkte Mundöffnung, anatomische Veränderungen in Gesicht, Larynx und Pharynx infolge der Muskelerkrankung, Kontrakturen im Bereich der HWS und eine verminderte Reklination ist das Risiko des schwierigen Atemwegs deutlich erhöht. In der internationalen Literatur wurden Intubationsprobleme bei Patienten mit UCMD beschrieben, wobei im fortgeschrittenen Krankheitsstadium häufig ein chirurgischer Atemweg besteht [13, 14]. Im vorliegenden Fall war 2018 komplikationslos eine Narkose zur Metallentfernung durchgeführt worden, die klinischen Verhältnisse hatten sich seitdem aber weiterverschlechtert. Der Algorithmus des erwartet schwierigen Atemwegs konnte bei der Patientin nicht vollständig eingehalten werden, da die kontinuierliche NIV eine fiberoptische Wachintubation deutlich erschwerte. Der Versuch musste wegen einer akuten kardiopulmonalen Dekompensation abgebrochen werden. Eine primäre Tracheotomie wurde von der Patientin abgelehnt, sodass sich als Alternative die Intubation in Narkose mittels Videolaryngoskop oder fiberoptisch bronchoskopisch über einen supraglottischen Atemweg oder eine Beatmungsmaske anbot. Es gibt die Empfehlung, während einer Narkose bei Patienten mit UCMD die Spontanatmung nach Möglichkeit zu erhalten [15, 16]. Diese Option bestand allerdings wegen der notwendigen Einlungenventilation für die VATS nicht. Zum Management des schwierigen Atemwegs bei thoraxchirurgischen Patienten sei auf einschlägige Übersichtsarbeiten verwiesen [17, 18]. Im vorliegenden Fall schlossen die anatomischen Verhältnisse die Platzierung eines Doppellumentubus aus, und die Einlungenventilation konnte komplikationslos mittels Bronchusblocker vorgenommen werden [19].

Die Auswahl der Anästhetika erfordert bei Patienten mit kongenitalen Muskelerkrankungen besondere Sorgfalt. Die Mehrzahl der Fallberichte bezieht sich allerdings auf Muskeldystrophien vom Typ Duchenne und Becker, wohingegen zur UCMD nur wenige Daten vorliegen [20]. In einer retrospektiven Zusammenstellung waren Succinylcholin und volatile Inhalationsanästhetika Risikofaktoren für bedeutsame Komplikationen, insbesondere bei nichtdiagnostizierten Muskelerkrankungen [20]. Succinylcholin ist generell bei Patienten mit Muskelerkrankungen kontraindiziert. Nichtdepolarisierende Muskelrelaxanzien (NDMR) können verwendet werden, wobei eine erhöhte Empfindlichkeit auf diese Substanzgruppe besteht und eine Antagonisierung mit Acetylcholinesteraseinhibitoren zu Komplikationen führen kann [21]. Zum Einsatz von Sugammadex bei UCMD liegen keine Daten vor [15]. Im vorliegenden Fall wurde sich gegen ein NDMR entschieden, da für die VATS im Regelfall eine tiefe Narkose ausreicht und die frühzeitige Rückkehr der Spontanatmung angestrebt wurde. Die Auswahl und Dosierung der Anästhetika hätten grundsätzlich eine Narkoseausleitung und Extubation im OP ermöglicht. Allerdings war die Spontanatmung unmittelbar postoperativ noch stark eingeschränkt, und das Risiko einer ungeplanten Reintubation sollte möglichst minimiert werden. Die schonende Entwöhnung von der Beatmung und Extubation auf der Intensivstation war daher die bessere Alternative.

Die postoperative Schmerztherapie ließ sich mit intravenösen Opioiden ohne Verschlechterung der Atmung suffizient durchführen. Grundsätzlich wäre auch eine thorakale Epiduralanästhesie infrage gekommen, im vorliegenden Fall infolge der ausgeprägten Skoliose, der Kontrakturen und der schwierigen Lagerung mit einem erhöhten Risiko verbunden gewesen. Bei thoraxchirurgischen Operationen wurden ultraschallgestützte Rumpfwandblockaden wie der thorakale Paravertebralblock, Interkostalblock, Pektoralisblockaden (PECS I, PECS II), Serratus anterior plane block oder M. errector spinae plane Block erfolgreich eingesetzt (Übersicht bei [22]). Diese Techniken ermöglichen eine suffiziente postoperative Schmerztherapie unter Vermeidung oder Reduktion intravenöser Opioide, allerdings liegen insbesondere zu Faszienblöcken bei einer Kollagen-assoziierten Myopathie keinerlei Daten vor.

Bei Patienten mit Muskelerkrankungen ist der Einsatz volatiler Inhalationsanästhetika kontrovers [23]. Traditionell werden sie als Auslöser einer malignen Hyperthermie (MH) betrachtet, neuere Studien weisen jedoch darauf hin, dass bei Dystrophinopathien keine tatsächliche MH getriggert wird, obwohl die Kardinalsymptome ähnlich sind [1]. Zudem sind Muskeldystrophien vom Typ Duchenne und Becker mit einer X‑chromosomalen Mutation assoziiert, während MH typischerweise auf Punktmutationen des Ryanodinrezeptor-kodierenden Gens *RYR1* auf Chromosom 19q13.2 sowie dem Protein-kodierenden Gen *CACNA1S* beruht und autosomal-dominant vererbt wird [1, 24]. Sevofluran und Desfluran wurden bei Patienten mit UCMD ohne Komplikationen eingesetzt, allerdings gibt es angesichts des seltenen Krankheitsbildes keine systematischen Studien [13–16]. Da volatile Inhalationsanästhetika bei Muskeldystrophien schwere Komplikationen wie Rhabdomyolyse, Hyperkaliämie und Asystolie auslösen können, wurde sich im vorliegenden Fall gegen den Einsatz entschieden und eine totale intravenöse Anästhesie unter BIS-Monitoring durchgeführt [25]. Allerdings ist zu beachten, dass bei mitochondrialen Myopathien und myotonen Syndromen der Einsatz von Propofol zu bedeutsamen Komplikationen führen kann [1]. Die Narkoseführung muss sich daher, soweit möglich, an dem zugrunde liegenden Krankheitsbild orientieren.
